# Impaired inhibitory reno-renal reflex responses in chronic kidney disease

**DOI:** 10.3389/fphys.2025.1544592

**Published:** 2025-04-09

**Authors:** Ahmed A. Rahman, Cara M. Hildreth, Phil Milliken, Sarah Hassan, Arun Sridhar, Jacqueline K. Phillips

**Affiliations:** ^1^ Sensory and Autonomic Neuroscience Laboratory, Macquarie Medical School, Faculty of Medicine, Health and Human Sciences, Macquarie University, Sydney, NSW, Australia; ^2^ Department of Pediatric Surgery, Massachusetts General Hospital, Harvard Medical School, Boston, MA, United States; ^3^ Galvani Bioelectronics, Stevenage, United Kingdom

**Keywords:** blood pressure, Capsaicin, chronic kidney disease, renal nerve, reno-renal reflex, sympathetic, sensory, splanchnic nerve

## Abstract

The renal afferent nerves serve as physiologic regulators of efferent renal sympathetic nerve activity (rSNA) as part of the inhibitory reno-renal reflex. Dysregulation of this reflex response may promote sympathoexcitation and subsequent hypertension under pathologic conditions such as chronic kidney disease (CKD). We have undertaken an in-depth characterization of reno-renal reflex function in CKD using an anesthetized rodent model with concurrent physiological outflows assessed. Using anesthetized male Lewis Polycystic Kidney (LPK) rats and normotensive Lewis controls, we investigated the cardiovascular [blood pressure (BP), heart rate (HR) and sympathetic responses (recorded from renal and splanchnic nerves (r/sSNA)] to renal capsaicin (50 µM) and direct electrical stimulation of the whole renal nerve. In Lewis rats, intra-pelvic renal capsaicin injection resulted in a depressor, bradycardic, and sympathoinhibitory response in sSNA with no significant change in rSNA. In contrast, the same stimulus led to a pressor and sympathoexcitatory response in the LPK group. In Lewis rats, low-intensity electrical stimulation (0.2 ms pulses, 15 μA, 2–40 Hz) of the renal nerve elicited a depressor response and bradycardia with concurrent sympathoexcitation (sSNA), whereas high-intensity (150 µA) stimulation induced a biphasic depressor/pressor response and tachycardia. In LPK rats, low-intensity renal nerve electrical stimulation triggered a biphasic depressor/pressor BP response, tachycardia, and sympathoexcitation. High-intensity stimulation similarly caused a biphasic depressor/pressor BP response and tachycardia. The magnitude of the sSNA response and both phases of the blood pressure response was higher in LPK compared to Lewis. All responses showed some degree of frequency dependency. Our results suggest the inhibitory reno-renal reflex is impaired in CKD, with dominance of excitatory reflex response. However, a depressor component remained that could be targeted using implantable neurotechnologies to lower blood pressure in CKD patients safely and effectively.

## 1 Introduction

The renal nerve contains both afferent and efferent nerve fibers. Under normal physiologic conditions, these fibers exert dual functions as part of the inhibitory reno-renal reflex. Afferent renal nerve activity exerts a tonic inhibitory effect on contralateral efferent renal sympathetic nerve activity (rSNA), resulting in compensatory natriuresis and diuresis in the contralateral kidney (Kopp, 2011a). Data suggests that this inhibitory reflex is tonically active (Protasoni et al., 1996; Ditting et al., 2012) and that this reflex may impact other sympathetic outflows (Saeki et al., 1988), mediated by supraspinal pre-autonomic regions (Solano-Flores et al., 1997). Within this loop, efferent rSNA facilitates increases in afferent renal nerve activity. Increased efferent rSNA is therefore a key stimulus in driving afferent renal nerve activity and constitutes an important negative feedback mechanism to maintain low levels of efferent rSNA. Long-term, this pathway facilitates homeostatic regulation of sodium and water balance, and subsequently blood pressure (BP), given that efferent rSNA decreases renal blood flow and glomerular filtration rate while increasing renal tubular sodium and water reabsorption and renin production (Johns et al., 2011). The negative feedback pathway, therefore, prevents excess renal sodium retention, which significantly impacts blood pressure. In addition to driving efferent rSNA, it is proposed that the reno-renal reflex may also drive other sympathetic outflows. Early studies in rabbits demonstrate a biphasic global SNA response following renal nerve stimulation with a corresponding depressor and pressor BP response (Saeki et al., 1988) and murine studies have demonstrated that stimulation of the renal afferent nerves produces a sympathetically-mediated pressor response (Ong et al., 2019).

A range of afferent renal sensory nerve responses are observed in different pathological conditions associated with increased sympathetic nervous system activity. In heart failure and hypertension rodent models, blunted afferent renal sensory nerve responsiveness is associated with unrestrained efferent rSNA and thus unopposed sympathetic activity to the kidneys, driving sodium and water retention (Kopp and Smith, 1996; Kopp et al., 2003; Kopp, 2011b). Similarily, impaired activation of the reno-renal reflex has been reported following renal damage secondary to acute kidney injury (Ma et al., 2002) and diabetes mellitus (Chien et al., 2000; Kopp et al., 2008). Conversely, heightened afferent renal nerve activity has been implicated in certain hypertensive models, where it drives sympathoexcitatory reflexes that exacerbate blood pressure elevation (Converse et al., 1992; Campese and Kogosov, 1995; Ye et al., 2002; Esler et al., 2024). Elevated afferent renal nerve activity could centrally amplify global SNA, including to the kidneys, and suggests that an excitatory reno-renal reflex may contribute to the pathophysiology of hypertension in specific disease states, potentially explaining the efficacy of renal denervation in its treatment (Phillips, 2005; Krum et al., 2011; Osborn and Banek, 2018).

In chronic kidney disease (CKD), evidence for aberrant sympathoexcitatory drive originating from the diseased kidneys includes studies in humans and animal models in which bilateral nephrectomy (Converse et al., 1992) or selective renal afferent denervation (Campese and Kogosov, 1995; Veiga et al., 2020) reduces BP and SNA. Notably, however, this is not consistent across all experimental models (Li et al., 2021), and there is limited data regarding the dysfunction of the reno-renal reflex in CKD.

In parallel with the increasing acceptance of renal denervation as a treatment for hypertension (Li and Phillips, 2022), there is a growing interest in electrical neuromodulation as an alternative therapeutic strategy. Unlike ablation, neuromodulation leverages existing reflex control pathways through precise neural stimulation, offering potential clinical benefits across a range of autonomic dysfunctions (Li et al., 2018; Lohmeier and Hall, 2019; Stavrakis et al., 2020; Hokanson et al., 2021; Stavrakis et al., 2024). If the inhibitory reno-renal reflex remains functional in disease states, it could be harnessed to elicit a depressor response and reduce BP. This is clinically relevant given the risks associated with renal denervation in patients with CKD, including impaired compensatory responses during hemorrhage, volume overload, and altered renal hemodynamics (Singh et al., 2021; Singh et al., 2022). Furthermore, disrupting an intact sympathoinhibitory reno-renal reflex through denervation may worsen hypertension in certain patients (Frame et al., 2019). Consequently, device-based neuromodulation strategies, such as spinal cord or nerve stimulation, are emerging as promising alternatives to selectively modulate reflex pathways without ablating nerve function. These approaches aim to improve BP regulation and address the limitations of pharmacological therapies and renal denervation. Comparable strategies have been proposed for managing cardiorenal syndrome (Zucker et al., 2023).

In the present study, we examined the integrity of the inhibitory reno-renal reflex in CKD using the Lewis Polycystic Kidney (LPK) rat model of CKD. The model exhibits early-onset and progressive kidney disease accompanied by hypertension from 6 weeks of age, closely mirroring key features of human CKD (Phillips et al., 2007). Notably, LPK rats demonstrate elevated rSNA under both conscious and anesthetised conditions, alongside impaired reflex autonomic function (Salman et al., 2015a; Yao et al., 2015). Given these characteristics, we hypothesised that the LPK would also exhibit an impaired reno-renal reflex response, consistent with evidence that diseased kidneys can drive aberrant sympathoexcitatory signalling as seen in other CKD contexts.

We used two different approaches to activate the reno-renal reflex. Firstly, we administered capsaicin to the renal pelvis as a direct sensory stimulus to drive afferent activation of the renal nerve measuring both the cardiovascular responses and effects on renal and splanchnic SNA (Gauthier et al., 2022). Capsaicin activates the renal afferent nerves through transient receptor potential vanilloid 1 (TRPV1) channels, which are expressed in the renal pelvic wall and sensory nerves, including unmyelinated C-fibres and myelinated Aδ fibres (Kopp, 2015). This activation induces the release of neuropeptides such as substance P and CGRP (Musolino et al., 2024). Direct TRPV1 channel activation has been shown to induce an inhibitory reno-renal reflex response resulting in increased afferent renal nerve activity (Banek et al., 2016) substance P release, and contralateral natriuresis (Kopp, 2011a). We hypothesized that this would elicit a classical mechanosensitive inhibitory reno-renal reflex in control Lewis rats (depressor and renal sympathoinhibitory response) but that this response would be either attenuated or reversed (pressor and sympathoexcitatory) in the setting of CKD. We also anticipated this sympathetic nerve response would be present in other sympathetic outflows. We then evaluated cardiovascular and SNA responses following direct electrical stimulation of the whole renal nerve at various intensities and frequencies to determine whether we could modulate the reno-renal reflex, either enhancing or reversing its cardiovascular and sympathetic effects in normal and diseased animals.

## 2 Methods

### 2.1 Animals

All animal experiments were performed in accordance with guidelines of the Australian Code of Practice for the Care and Use of Animals for Scientific Purposes under protocols approved by the Animal Ethics Committee of Macquarie University, Sydney, Australia. Male Lewis and LPK rats aged 12–14 weeks (n = 36) were used for the experiments. Animals had free access to a standard rat diet and water *ad libitum* and were group housed in a temperature-controlled environment with a 12-h day/night cycle. Animals were allowed to acclimatize to the facility for a minimum of 7 days before experimental manipulations.

### 2.2 Electrophysiological experiments

#### 2.2.1 Surgical preparation

Animals were anaesthetized with urethane (1.2–1.4 g/kg, i.p.) with supplemental doses (30–40 mg, i.v.) given as needed if nociceptive stimuli (tested every 15 min) caused a change in mean arterial pressure (MAP) of more than 10 mmHg. Rectal temperature was monitored and maintained at ∼36.5°C with a thermostatically controlled heating pad (Harvard Apparatus, Holliston, MA, United States) and infrared heat lamp. The right femoral artery and vein were cannulated with polyethylene tubing (internal diameter = 0.58 mm; outer diameter = 0.96 mm) to measure arterial pressure and administer drugs and necessary fluids, respectively. The trachea was cannulated to enable artificial ventilation, and a 3-lead electrocardiogram was fitted. Heart rate (HR) was determined from the BP recordings. The left greater splanchnic nerve and left or right renal nerve were isolated. Nerves were left intact if used for stimulation, or ligated with silk sutures, and transected distally to permit recording of efferent splanchnic sympathetic nerve activity (sSNA) and renal sympathetic nerve activity (rSNA), respectively. In one cohort of animals (n = 3 LPK; 3 Lewis), the central end of the left renal nerve was cut to eliminate efferent nerve traffic allowing the recording of baseline afferent renal nerve discharge. Rats were secured in a stereotaxic frame, vagotomized unless otherwise stated, paralysed (pancuronium bromide; 0.8 mg initially, then 0.4 mg/h), and artificially ventilated with oxygen-enriched room air. End-tidal CO_2_ was monitored and maintained between 4.0% and 4.5% (Capstar 100, CWE Inc., Ardmore, PA, United States). Arterial blood gas specimens were analysed to maintain pH at 7.35 ± 7.45 (pH 7.4 ± 0.03; PaCO_2_ 40.4 ± 0.9) using an electrolyte and blood gas analyser (IDEXX Vetstat, West brook, ME, United States). Animals were infused with 5% glucose in water (1.0–2.0 mL/h) to ensure hydration. Nerve recordings were obtained with bipolar silver wire electrodes. The neurograms were amplified (x10 000, CWE Inc.), bandpass filtered (0.1–2 kHz), sampled at 3 kHz (1,401 plus, CED Ltd., Cambridge, United Kingdom), and recorded on computer using Spike2 software (v7.1, CED Ltd.).

#### 2.2.2 Capsaicin stimulation

In this cohort of animals (n = 6 LPK; 5 Lewis), the right renal pelvis was cannulated with a 32G triple-lumen catheter (Part Number: 0040EO; ReCathCo, Allison Park, PA, United States) for intrapelvic bolus injection and the contralateral left rSNA and sSNA were used for recordings. Capsaicin (dose: 50 μM, 300 μL volume) was injected once over 30–40 s as part of a standardized administration protocol to ensure consistent delivery and avoid transient bolus-induced effects on blood volume. Mean arterial blood pressure, diastolic blood pressure (DBP), systolic blood pressure (SBP), HR and SNA were assessed at peak response to capsaicin, which was seen consistently within 60 s of administration in all animals

#### 2.2.3 Electrical stimulation

In group 1 (n = 5 Lewis, minimum n = 3 LPK), the left renal nerve (intact, and therefore activating both afferent and efferent components) was isolated for stimulation, and ipsilateral recordings from the left splanchnic nerve were obtained using custom-made bipolar electrodes. The polarity of the stimulation electrode was tested in both normal (cathode towards the kidney) and reversed (cathode towards the spinal cord) configurations for each animal to account for potential differences in nerve activation. Cathodal stimulation facilitates neuronal depolarisation and action potential generation, whereas anodal stimulation has the potential to hyperpolarise neurons, potentially inducing an anodal block that may bias efferent versus afferent nerve activation (Ahmed et al., 2020). No significant polarity bias was observed in the measured endpoints (Supplementary Figures S1, S2). In group 2 (minimum n = 3 per group), the left renal nerve (intact, containing both afferent and efferent components) was isolated and stimulated using cuff electrodes [sling and tunnel (150 μm; Cortec, Freiburg, Germany)] and recordings were obtained from the left (ipsilateral) splanchnic nerve. In group 3 (minimum n = 3 per group), the right renal nerve (intact, containing both afferent and efferent components) was isolated and stimulated using cuff electrodes and recordings were obtained from the contralateral left splanchnic nerve. These experiments were undertaken for investigation into potential laterality differences, as have been described for the aortic depressor nerve (Salman et al., 2020) noting there was no significant effect of electrode placement for the measured parameters (Supplementary Figures S3–S5). In these experiments, animals were non-vagotomised to ensure that the sympathetic responses recorded reflected a fully functioning autonomic nervous system. Stimulation (monophasic 0.2 ms pulses at 15 s train) current was generated using an Iso-flex (AMPI, Jerusalem, Israel) stimulator. In each animal, stimulation at a range of intensities and frequencies was performed. Low-intensity was set at 15 μA, and high-intensity was set at 150 µA. These stimulation thresholds were chosen based on observed physiological responses in pilot studies, with thresholds determined by titrating current intensity to achieve reproducible responses while minimizing the risk of tissue damage. Frequency was tested at 2, 5, 10, 20 and 40 Hz (in random order for each animal).

#### 2.2.4 Data acquisition

Neurograms were rectified and smoothed (splanchnic nerve 1 s time constant; renal nerve, 50 ms). Minimum background activity after death was taken as zero, and this value was subtracted from nerve activity before analysis with offline software (Spike 2 version 7). Baseline SNA values were obtained by averaging 60 s of data 5 min before capsaicin injection or electrical stimulation. Maximum changes were expressed as absolute or percentage changes from baseline values.

#### 2.2.5 Data analysis

Grouped data are expressed as mean ± SEM. Statistical analysis was conducted with GraphPad Prism (version 7.0) (Graph-Pad, La Jolla, CA, United States). Analysis of baseline data was undertaken using an unpaired t-test comparing strains. Analysis of SBP, sSNA, and/or rSNA responses to experimental stimuli was undertaken using repeated measures Two-way or One-way ANOVA (or mixed effects if values were missing) as appropriate. Depressor and pressor SBP, and bradycardic and tachycardic HR responses, respectively, were analysed separately. Data was tested for equal variances before analysis to confirm the appropriate *post hoc* method. For capsaicin experiments, the matching values were before and after capsaicin injection. The effect of strain was also assessed. For Group 1, the effect of frequency and polarity were initially used as matching values as both parameters were varied in individual animals. Polarity was only a significant variable for one component of the data set (Supplementary Figures S1, S2). As such, both sets of data were included in *post hoc* analysis to determine the effect of strain and frequency in a second analysis of the data. For studies examining frequency and recording electrode placement (Groups 2 and 3), frequency was used as the matching value. Recording electrode placement (ipsilateral or contralateral) was not a significant variable for any of the parameters measured (Supplementary Figures S3–S5), and the data was combined to assess the effect of strain and frequency in a second analysis of the data. Bonferroni *post hoc* analysis was performed as indicated by the ANOVA results. P < 0.05 was considered significant. Analysis was undertaken to compare the responses when using either a bipolar or cuff-stimulating electrode (Supplementary Material). Frequency and electrode type were used as the fixed effects within strain for two-way ANOVA analysis. Overall, the responses were in the same direction regardless of electrode type and of very similar magnitude within strain. The data presented, therefore, was obtained using bipolar stimulating electrodes, and the data obtained using the cuff electrodes is provided as confirmatory data within Supplementary Figures S6, S7.

## 3 Results

### 3.1 Afferent renal nerve activity

Baseline afferent renal nerve activity recordings revealed a significant difference between the two groups. The mean afferent renal nerve activity in Lewis rats was 0.65 ± 0.2 μV, while in LPK rats, it was 1.6 ± 0.2 μV (P = 0.0183). Representative trace recordings of raw data for the afferent renal nerve activity are provided in Supplementary Figure S8.

### 3.2 Cardiovascular hemodynamic and sympathetic responses to intrarenal capsaicin injection in Lewis and LPK animals

**FIGURE 1:**
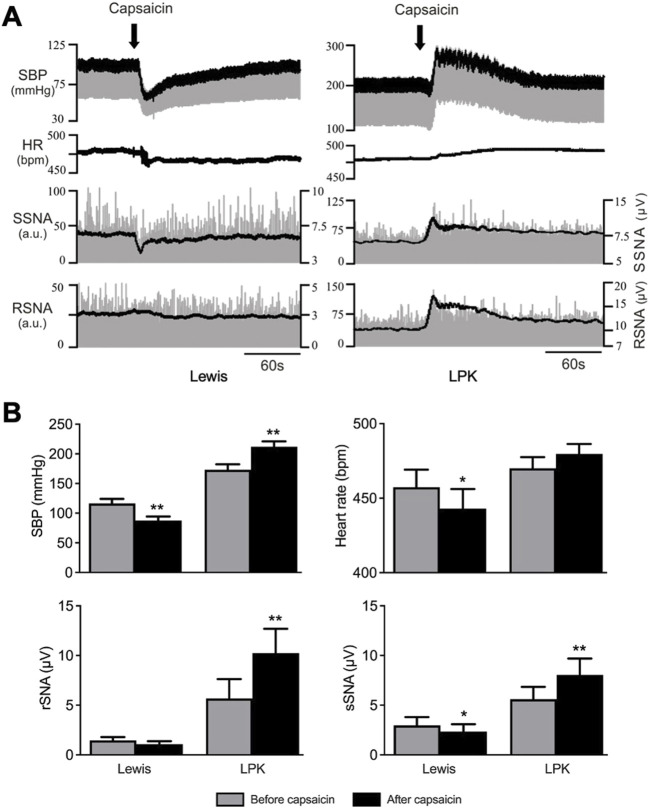
Cardiovascular and SNA responses to renal pelvic capsaicin injection Effect of renal pelvic capsaicin (50 μM) on systolic blood pressure (SBP), heart rate (HR) and contralateral splanchnic (sSNA) and renal sympathetic nerve activity (rSNA). **(A)** Representative trace of data from a Lewis and LPK recording of SBP (mmHg), HR (bpm) and sSNA/rSNA [arbitrary units (a.u.)] before and after injection of capsaicin. SBP is superimposed over raw blood pressure recording and rectified and integrated SNA is superimposed over raw SNA data. Scale on left side is for raw SNA, scale on right is for rectified and integrated sSNA. Note scales for SBP and SNA are different for Lewis and LPK animals allowing depiction of different magnitude responses in the two strains. Scale bar represents 60 s for each panel. **(B)** Grouped data showing effects of capsaicin injection on SBP, HR, contralateral rSNA and sSNA in Lewis and LPK animals illustrated as before and after capsaicin injection. Values are expressed as mean ± SEM. N = 6 for each strain for SBP, sSNA and HR for LPK animals, n = 5 for all other groups. **P < 0.01, *P < 0.05 before vs. after capsaicin. bpm, beats per minute.

Baseline SBP recordings under anesthesia before capsaicin injection confirmed the hypertensive phenotype of the LPK strain as compared to the control Lewis strain (SBP: P = 0.0001). There was no difference in HR, rSNA, or sSNA between the two strains at baseline. Figure 1A illustrates trace recordings of cardiovascular and contralateral SNA responses to local capsaicin injection into the right renal pelvis. In the Lewis rat, capsaicin injection induced a significant depressor response accompanied by bradycardia and sympathoinhibition in contralateral sSNA but not in rSNA (Figure 1B). In the LPK rat, intra pelvic capsaicin elicited a significant pressor response with concurrent sympathoexcitatory responses in both contralateral sSNA and rSNA (Figure 1B). There was no significant change in HR in the LPK animals.

### 3.3 Cardiovascular and sympathetic responses to electrical stimulation of the renal nerve in Lewis and LPK animals

#### 3.3.1 Effects of low-intensity stimulation (15 µA) via bipolar electrode on ipsilateral sympathetic activity and cardiovascular hemodynamics in Lewis and LPK animals

**FIGURE 2:**
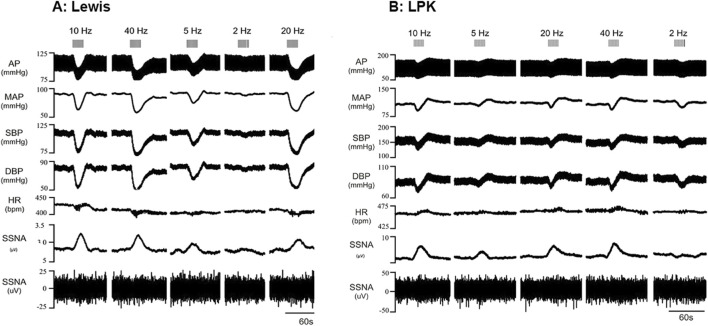
Effects of low-intensity stimulation of the renal nerve using a bipolar electrode on cardiovascular parameters and sympathetic activity in Lewis and LPK animals Effects of left renal nerve stimulation (15uA) at different Hz (2, 5, 10, 20 and 40) on blood pressure and sympathetic nerve activity in a Lewis **(A)** and LPK **(B)** rat. Representative trace of data show recording of full arterial pressure trace (AP), mean arterial pressure (MAP), systolic blood pressure (SBP), diastolic blood pressure (DBP), heart rate (HR) and left splanchnic sympathetic nerve activity (ipsilateral sSNA), with both rectified and integrated, and raw data provided. Response to stimulation of the left renal nerve at the different frequencies is as indicated by grey bars at the top of the panel. Note the scale for Lewis and LPK animals varies for the individual parameters due to the difference in the range of the response in the different strains.

The left renal nerve was stimulated using a custom-made bipolar electrode at 15 μA at various frequencies (2, 5, 10, 20 and 40 Hz). Representative traces of cardiovascular and ipsilateral SNA responses in the left splanchnic nerve to electrical stimulation at different frequencies are illustrated in Figure 2 (A: Lewis, B: LPK). Expanded traces of the raw data for sSNA are provided in Supplementary Figure S9. Statistical results for *post hoc* frequency analysis are provided in Supplementary Data S1.

**FIGURE 3:**
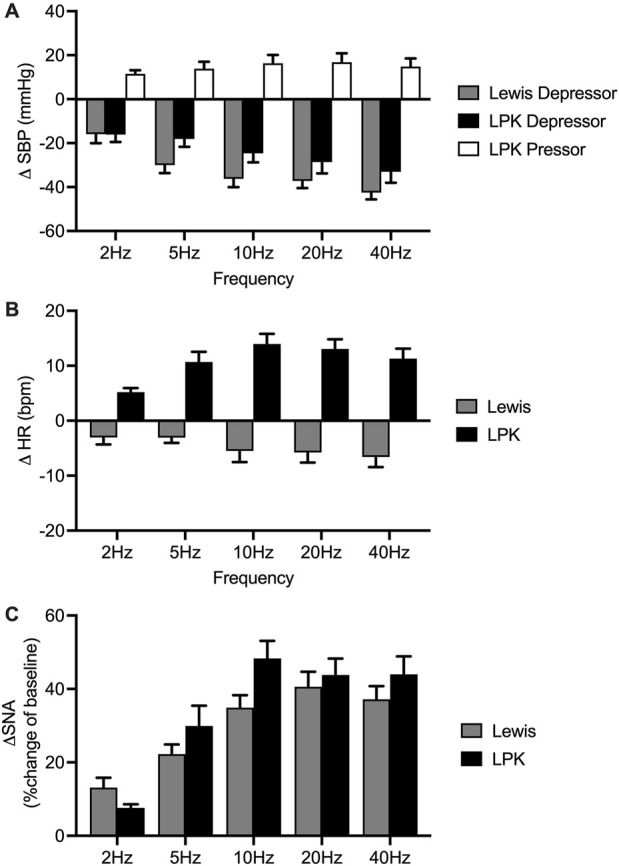
Group effects of low-intensity stimulation of the renal nerve using a bipolar electrode on cardiovascular parameters and sympathetic activity in Lewis and LPK animals. Grouped data showing peak systolic blood pressure (SBP: depressor and pressor), heart rate (HR: (bradycardia and tachycardia) and left splanchnic sympathetic nerve activity (sSNA) produced by left renal nerve stimulation (15uA) at different Hz (2, 5, 10, 20 and 40) in Lewis and LPK rats. Effects are shown as absolute change [**(A)**: ΔSBP, **(B)**: ΔHR] or percentage [**(C)**: ΔsSNA] changes from respective baseline value. Values are expressed as mean ± SEM and are calculated using both the response under normal and reverse polarity for each animal (n = 6 LPK, n ≥ 8 Lewis).

In the Lewis animals, low-intensity stimulation caused a depressor response, bradycardia and sympathoexcitation (Figure 3). There was a significant effect of frequency on the depressor (P < 0.001; Figure 3A) and sympathoexcitatory responses (P < 0.0001; Figure 3C). In the Lewis animals, the depressor response reached a peak and plateaued at 5–10 Hz. The sSNA response reached a peak level and plateaued at 10 Hz. There was no frequency-dependent effect on HR (P = 0.0824, Figure 3B).

In the LPK rats, low-intensity renal nerve stimulation induced a biphasic response on BP (depressor followed by pressor response) with tachycardia and sympathoexcitation (Figure 3). Depressor and pressor responses were analysed separately. There was a frequency-dependent effect on the depressor response (P = 0.002: Figure 3A) which peaked and then plateaued across the 5–10 Hz responses. There was no frequency dependent effect on the pressor response (P = 0.1750). There was also a frequency-dependent effect on HR (P < 0.0001; Figure 3B), with a peak effect that plateaued at 10 Hz. The sympathoexcitatory response was frequency-dependent (P < 0.0001; Figure 3C), with a peak effect that plateaued at 10 Hz.

Cardiovascular (SBP and HR) responses at low frequency were not compared between strains due to the different type of responses seen in the two strains. When comparing sSNA activity following bipolar electrical stimulation at 15uA, no statistically significant difference in the magnitude of the sympathoexcitatory response between strains was evident (P = 0.2888; Figure 3C).

#### 3.3.2 Effects of high-intensity stimulation (150 µA) using bipolar electrode on ipsilateral sympathetic activity and cardiovascular hemodynamics in Lewis and LPK animals

**FIGURE 4:**
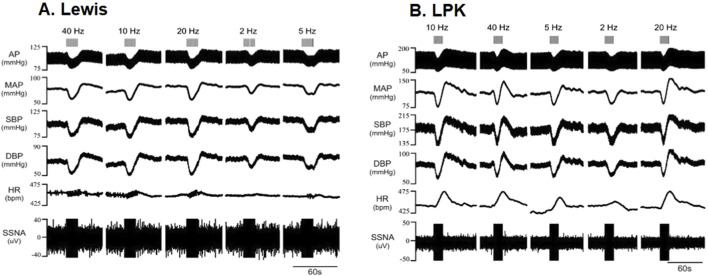
Effects of high-intensity stimulation of the renal nerve using a bipolar electrode on cardiovascular parameters and sympathetic activity in Lewis and LPK animals Effects of left renal nerve stimulation (150 uA) at different Hz (2, 5, 10, 20 and 40) on blood pressure and sympathetic nerve activity in a Lewis **(A)** and LPK **(B)** rat. Representative trace of data show recording of full arterial pressure trace (AP), mean arterial pressure (MAP), systolic blood pressure (SBP), diastolic blood pressure (DBP), heart rate (HR) and left splanchnic sympathetic nerve activity (ipsilateral sSNA). Only raw data is provided for sSNA as noise during period of stimulation prevented determination of sSNA change. Response to stimulation of the left renal nerve at the different frequencies is as indicated by grey bars at the top of the panel. Note the scale for Lewis and LPK animals varies for the individual parameters due to the difference in the range of the response in the different strains.

**FIGURE 5:**
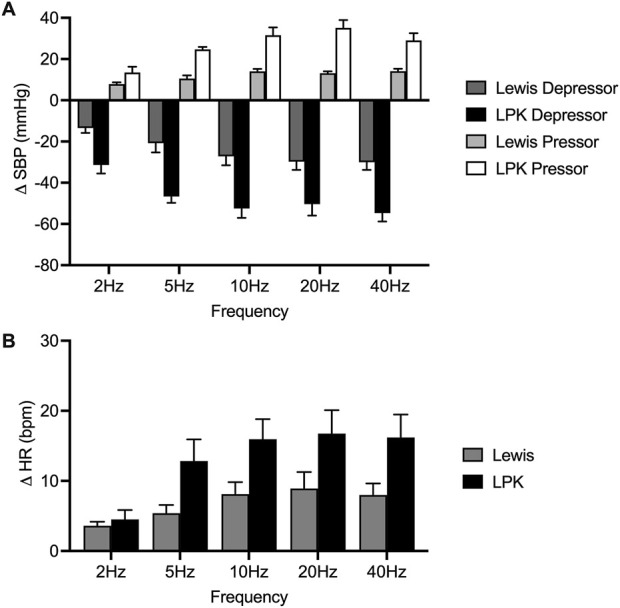
Grouped effects of high-intensity stimulation of the renal nerve using a bipolar electrode on cardiovascular parameters in Lewis and LPK animals. Grouped data showing peak systolic blood pressure (SBP: depressor and pressor) and heart rate (HR: bradycardia and tachycardia) responses produced by left renal nerve stimulation (150uA) using a bipolar electrode at different Hz (2, 5, 10, 20 and 40) in Lewis and LPK rats. Effects are shown as absolute change [**(A)**: DSBP, **(B)**: DHR] from respective baseline value. Values are expressed as mean ± SEM and are calculated using both the response under normal and reverse polarity for each animal (n = 3 LPK, n = 5 Lewis).

The left renal nerve was stimulated using a custom-made bipolar electrode at 150 μA at different frequencies (2, 5, 10, 20 and 40 Hz). Representative traces following stimulation at various frequencies are illustrated in Figure 4 (A:Lewis, B: LPK), noting that noise during period of stimulation prevented determination of ipsilateral sSNA change. Grouped data are shown in Figure 5. Statistical results for *post hoc* frequency analysis are provided in Supplementary Data S1.

In the Lewis animals, high-intensity stimulation triggered a biphasic BP response (depressor/pressor) and tachycardia. Noise during the stimulus precluded assessing changes in sSNA. The depressor response was significantly impacted by frequency (P < 0.001; Figure 5A), with the depressor response peaking and reaching a plateau at 5–10 Hz. Post-hoc analysis did not identify any differences between the pressor responses in the Lewis at any frequency. Similarly for HR, *post hoc* analysis did not identify any changes in HR between frequencies (Figure 5B).

In LPK rats, high-intensity renal nerve stimulation induced a biphasic BP response (depressor/pressor) with concurrent tachycardia. All parameters were significantly impacted by frequency (P < 0.05). The depressor response reached a peak and plateau at 5 Hz (Figure 5A). The pressor response peaked and reached a plateau at 5–10 Hz. The tachycardic HR response in the LPK reached a peak and plateau at 5 Hz (Figure 5B).

When comparing experimental groups, LPK animals exhibited significantly greater magnitude changes in both the depressor and pressor phases of the BP response (both P < 0.05; Figures 5A,B). Additionally, high-intensity stimulation triggered a greater increase in HR in the LPK group (P < 0.005; Figure 5C).

## 4 Discussion

Despite the predicted role of afferent renal nerve activity as a driver of increased BP in CKD, and clinical data demonstrating the safety and the BP-lowering efficacy of renal denervation (Barbato et al., 2023) there is a notable gap in the literature examining perturbations, if any, to the reno-renal reflex in CKD. In the present study we therefore investigated the integrity and functionality of the reno-renal reflex in CKD using the LPK rat model, where we have previously shown an elevated BP and SNA compared to healthy Lewis rats (Salman et al., 2014; Yao et al., 2015), utilizing two distinct methods to activate the reflex pathway.

Herein, we have shown that under our experimental conditions, capsaicin drives a classical reno-renal reflex in the Lewis rat, eliciting a depressor response with bradycardia and sympathoinhibition in both sSNA and rSNA. In contrast, we evoked the inverse response in the LPK rat model of CKD, as demonstrated by a pressor response accompanied by sympathoexcitation, suggesting a significant change in the reno-renal reflex in the diseased kidney.

Low-intensity electrical stimulation of the renal nerve, either with a bipolar electrode or cuff electrode, elicited a decrease in BP and HR with concurrent sympathoexcitation in Lewis animals. In contrast, LPK rats demonstrated a biphasic (depressor/pressor) BP response, tachycardia and sympathoexcitation to the same stimulus. Of note is that high-intensity renal nerve stimulation in Lewis rats mirrored the response demonstrated by LPK rats. Other important observations include that the responses to these stimuli all demonstrated some degree of frequency dependence and electrode polarity did not make a significant impact on the response type or magnitude. We also demonstrate that renal nerve stimulation elicited comparable sympathetic responses in both the ipsilateral and contralateral nerves, indicating no laterality in the sympathetic reflex response.

### 4.1 Afferent renal nerve activity

Our study revealed elevated baseline afferent renal nerve activity in the LPK rat model, supporting the concept of heightened afferent signaling from diseased kidneys (Phillips, 2005). This observation aligns with prior findings in other pathophysiological models, including DOCA-salt hypertensive rats (Banek et al., 2016) and another genetic model of CKD (Gauthier et al., 2022). These findings suggest that resting afferent renal nerve activity can shift and therefore may contribute to reno-renal reflex dysfunction in CKD.

### 4.2 Response to capsaicin

Under normal physiologic conditions, the inhibitory reno-renal reflex is thought to be mediated by activation of renal mechanosensory nerves. In our study, we investigated this reflex using capsaicin as a direct activator of TRPV1 channels. Consistent with renal afferent TRPV1-mediated pathways (Kopp, 2015; Banek et al., 2016; Gauthier et al., 2022), our experiments in the normotensive Lewis rat demonstrated reductions in arterial pressure, HR, and contralateral SNA (both splanchnic and renal) following capsaicin injection into the renal pelvis. Other studies examining the response to pelvic administration of capsaicin in normal animals have similarly shown a sympathoinhibitory response (Kopp and Smith, 1996; Ditting et al., 2012). This is distinct from studies where capsaicin has been administered intra renally via the renal artery, where normotensive animals instead demonstrate a sympathoexcitatory and vasopressor response (DeLalio and Stocker, 2021; Ye et al., 2021).

In direct contrast to the response in the control animals, intrapelvic capsaicin injection in the CKD animals triggered an increase in BP and contralateral SNA and no change in HR. The maintenance of HR, distinct from the decrease in the controls, maybe driven by compensatory mechanisms to maintain hemodynamic stability, noting the marked increased in BP. Overall, this is consistent with our hypothesis of an impaired inhibitory reno-renal reflex and specifically the dominance of an excitatory response, not dissimilar to that demonstrated by Kopp et al. in the two-kidney, one-clip hypertensive rat (Kopp and Buckley-Bleiler, 1989). A predominantly excitatory reno-renal reflex in CKD may represent a shift from a mechanosensory-stimulated response (inhibitory reflex) to a predominantly chemoreceptor-stimulated one (excitatory reflex), mediated by the proposed R1 and R2 chemoreceptors. The renal R1 and R2 chemoreceptors represent distinct populations of sensory nerve endings in the kidney that respond to chemical rather than mechanical stimuli (Rogenes, 1982). R1 chemoreceptors are thought to be activated by renal ischemia, while R2 chemoreceptors are activated by both ischemia and changes in urinary ionic composition such as backflow of concentrated urine into the renal pelvis (Recordati et al., 1978; Recordati et al., 1982; Chien et al., 2000). Stimulation of R1 and R2 chemoreceptors elicits an excitatory renorenal reflex that increases efferent renal sympathetic nerve activity to both the ipsilateral and contralateral kidneys, exerting their effects via supraspinal pathways, although some spinal integration may occur (Recordati et al., 1978; Recordati et al., 1982).

The mechanism by which the balance between mechanoreceptor-mediated inhibitory reflexes and chemoreceptor-mediated excitatory reflexes shifts in the diseased kidney could result from multiple factors along the reflex pathway. As discussed, increased basal afferent tone may play a role, consistent with other models of kidney disease. Renal sensory receptor dysfunction is also a possibility. In early diabetes, both R2 chemoreceptor and ureteropelvic mechanoreceptor responses are diminished due to pathological changes in the kidney including histamine-induced chemoreceptor sensitisation and collagen-related mechanoreceptor dysfunction (Chien et al., 2000). In LPK rats, advanced collagen deposition and fibrosis are present (Phillips et al., 2007) and may similarly alter receptor sensitivity and the balance of afferent signalling. The response may also be influenced by differences in basal sympathetic tone, autonomic function, and cardiovascular physiology. LPK rats exhibit elevated baseline blood pressure and rSNA (Salman et al., 2015a; Yao et al., 2015), alongside impaired autonomic reflexes. These include deficits in baroreflex control of HR and rSNA (Salman et al., 2015b) and blunted vagal afferent-mediated reflex sympathoinhibition (Salman et al., 2017). Such central dysregulation of autonomic outflows may amplify a sympathoexcitatory response to renal sensory afferent input. Additionally, vascular remodelling (Ng et al., 2011) and dysregulated renin-angiotensin-aldosterone system activity (Phillips et al., 2007) in LPK rats could enhance sympathoexcitation through reduced buffering capacity. In contrast, Lewis rats may possess more effective counter-regulatory mechanisms, contributing to their inhibitory renorenal reflex response.

### 4.3 Response to nerve stimulation

In the second series of experiments, we investigated the response to electrical stimulation of the renal nerve in both control and diseased animals. These experiments are fundamental to a determination as to whether electrical neuromodulation could enhance the inhibitory reno-renal reflex and reduce BP in the context of CKD. Previous studies in normotensive animals report disparate changes to BP and SNA in response to renal nerve stimulation. In the early work of Saeki et al., (1988), they describe three responses. In the majority of animals, there was a sympathoinhibition and decrease in BP, however, they also observed animals that had subsequent period of sympathoexcitation, and in turn a biphasic BP response (depressor/pressor), as well as animals that demonstrated sympathoexcitation and a pressor response. More recently, studies in mice using parameters comparable to our high-intensity parameters demonstrated frequency-dependent increases in BP as well as increased SNA to multiple nerve beds (Ong et al., 2019). Differential activation of myelinated (A) and unmyelinated (C) afferent fibres are though to contribute to variable effects reported as well as the subsequent engagement of distinct central neural pathways (Saeki et al., 1988; Zheng and Patel, 2017).

In this study, in the healthy Lewis rat, low-intensity stimulation of the renal nerve at various frequencies and polarities induced a depressor response and bradycardia with concurrent sympathoexcitation, in contrast to the sympathoinhibition observed after renal nerve stimulation with capsaicin. The different SNA responses to capsaicin vs. low-intensity nerve stimulation may be explained by recruitment of additional afferent inputs, including those likely responsible for the excitatory reno-renal seen in response intrarenal capsaicin (DeLalio and Stocker, 2021; Ye et al., 2021), as well as likely co-activation of the baroreflex as part of an integrated reflex response. In contrast, high-intensity stimulation induced a biphasic depressor/pressor response and tachycardia (noting we were unable to measure SNA due to noise during the stimulation period). A potential explanation for this is a threshold effect is reached at the higher intensity, resulting in differential activation of A and C afferent fibres as noted above.

The significance of the biphasic response in the control animals under high-intensity stimulation has greater relevance when considering the response to direct electrical stimulation of the renal nerve in the CKD animals. They responded with a biphasic depressor/pressor response with simultaneous tachycardia and sympathoexcitation under both low and high-intensity parameters. Considering the threshold effect postulated above, these results suggest that the threshold has been significantly reduced in the LPK, whereby low-intensity stimulation parameters trigger the biphasic BP response. This notion supported further by the finding that the BP and HR responses under high-intensity stimulation parameters were greater in the LPK than in the control animals. This altered threshold could be linked to CKD-related pathological changes, noting that in the DOCA-salt model, inflammation was shown to contribute to the heightened afferent renal nerve activity and sympathoexcitation (Banek et al., 2016) and inflammation is a key feature of the LPK model (Phillips et al., 2007). Further, as discussed with reference to the response to capsaicin, the dominance of excitatory responses seen in the LPK may be contributed to by changes in the complex interplay between inhibitory and excitatory components along the reflex pathway including altered thresholds and recruitment of distinct neuronal circuits (Johns et al., 2011).

### 4.4 Limitations

We acknowledge that the current study has several limitations. As noted above, experiments were conducted using an anesthetized animal model, exclusively involving male subjects. Research by DeLalio and Stocker, (2021) has demonstrated that anesthesia, and to a lesser extent sex, can attenuate sympathetic efferent and hemodynamic responses to intra-arterial renal chemosensory and renal pelvis mechanosensory stimuli. Our use and choice of type of anesthetic may therefore have obscured the full physiological response, and the exclusion of female subjects may have overlooked potential sex-specific differences in autonomic function, as previously identified in both preclinical animal models (Salman et al., 2015b) and human CKD patients (Zanuzzi et al., 2024). Additionally, differences in BP and HR responses between anesthetised and conscious states have been reported. While anesthesia was necessary for reliable SNA recordings in this study, future research could address this limitation by employing chronic conscious models with implanted electrodes tethered to external stimulators and telemetry systems for BP/HR measurements (Salman et al., 2015a). Further, groups had small numbers which may limit statistical power, however, power calculations ensured sufficient sensitivity to detect robust changes in BP/HR/SNA. Future studies could benefit from larger cohorts and diversifying the experimental conditions to improve generalizability and further validate the findings.

### 4.5 Clinical translation

Selective neuromodulation has demonstrated efficacy in lowering BP through reflex pathways, such as carotid baroreceptor activation (Lohmeier and Hall, 2019). In CKD, targeting preserved depressor responses via the renorenal reflex represents an emerging therapeutic strategy with significant potential. However, risks such as transient BP elevations, arrhythmias, and altered renal hemodynamics—particularly relevant in advanced CKD—highlight the need for cautious translation to clinical practice.

This study provides foundational proof of concept that renal nerve stimulation can engage the renorenal reflex to elicit depressor responses even in diseased kidneys. We used both custom-made bipolar electrodes and extra-neural cuff electrodes, the latter representing a significant step towards implantable neurotechnologies currently being developed for autonomic disorders (Larson and Meng, 2020). Cuff electrodes may provide a bespoke neural interface and more targeted approach for modulating nerve activity while minimising off-target effects because they enable stable and consistent electrical stimulation delivery to the nerve. This is due to their capacity to isolate the electrical current to the nerve, avoiding unintended spread to surrounding tissues (i.e., resulting in fasciculation) and standardizing charge density delivery due to fixed electrode pitch dimensions (Popovic et al., 1991). Future studies should prioritize conscious animal models to refine stimulation parameters including exploration of frequency dependency, mitigate off-target effects, and validate translatable neuromodulation strategies. Parameter optimization in these models will identify therapeutic stimulation ranges while eliminating anesthetic confounders and ensuring safety.

## 5 Conclusion

Our study demonstrates a significant shift in the reno-renal reflex in CKD, transitioning from a predominantly inhibitory response to an excitatory one, likely contributing to elevated BP. Given that the same renal afferent nerve fibres can elicit both inhibitory (BP lowering) and excitatory (BP raising) responses, our findings indicate that targeted therapeutic modulation of this reflex to achieve effective BP control may be feasible.

Future research exploring technologies like fibre-specific stimulation, optimization of stimulation parameters, and novel electrode configurations could harness the inhibitory component of the reno-renal reflex to safely reduce BP in CKD. Such neuromodulation approaches could offer valuable alternatives to current treatments, providing more precise control of hypertension and potentially improving cardiovascular and renal outcomes.

## Data Availability

The original contributions presented in the study are included in the article/Supplementary Material, further inquiries can be directed to the corresponding author.
