# Supplementary material for: Impaired inhibitory reno-renal reflex responses in chronic kidney disease
Source: Front Physiol. 2025 Apr 9;16:1544592. doi: 10.3389/fphys.2025.1544592 (PMC12014541; doi:10.3389/fphys.2025.1544592)
Supplement: Supplementary file 1 [file DataSheet1.pdf]

Impaired reno-renal reflex responses in chronic kidney disease

Supplementary Material

Ahmed A Rahman[1,2], Cara M Hildreth[1], Phil Milliken[3], Sarah Hassan[3], Arun Sridhar[3], Jacqueline K Phillips[1*]

[1] Sensory and Autonomic Neuroscience Laboratory, Macquarie Medical School, Faculty of Medicine, Health and Human Sciences, Macquarie University, Sydney, NSW, Australia

[2] Department of Pediatric Surgery, Massachusetts General Hospital, Harvard Medical School, MA, USA.

[3] Galvani Bioelectronics, Stevenage, SG1 2NY, UK

*Corresponding Author: jacqueline.phillips@mq.edu.au

Table of Contents

Table of Contents 1

Supplementary Figure 1: Comparison of polarity (15μA)...2

Supplementary Figure 2: Comparison of polarity (150μA)..3

Supplementary Figure 3: Comparison of recording cuff electrode placement (ipsilateral or contralateral to SNA bipolar recording electrode) on cardiovascular parameters in Lewis animals. 4

Supplementary Figure 4: Comparison of recording cuff electrode placement (ipsilateral or contralateral to SNA bipolar recording electrode) on cardiovascular parameters in LPK animals 5

Supplementary Figure 5: Comparison of recording cuff electrode placement (ipsilateral or contralateral to SNA bipolar recording electrode) on sympathetic response in Lewis and LPK animals 6

Supplementary Analysis: Comparison of responses using bipolar vs. cuff electrodes...7

Supplementary Figure 6: Grouped effects of low-intensity stimulation of the renal nerve using a cuff electrode on cardiovascular parameters and sympathetic activity in Lewis and LPK animals 8

Supplementary Figure 7: Grouped effects of high-intensity stimulation of the renal nerve using a cuff electrode on cardiovascular parameters in Lewis and LPK animals...9

Supplementary Figure 8: Baseline afferent renal nerve activity 10

Supplementary Figure 9: Response of splanchnic sympathetic nerve to renal nerve stimulation 11

Supplementary Figure 1: Comparison of polarity (15µA)

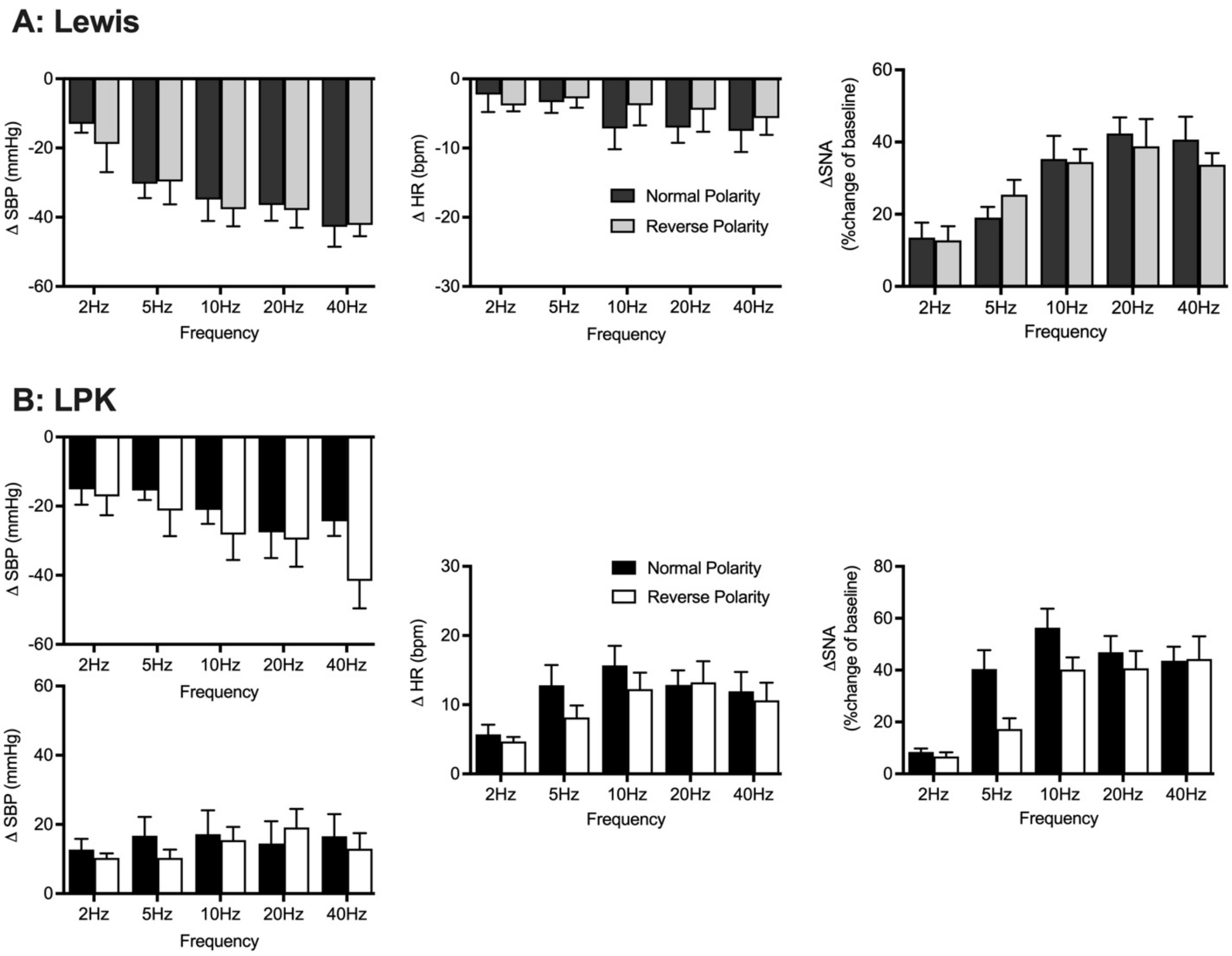

Group data showing comparison of left renal afferent nerve stimulation (15uA) at different Hz (2, 5, 10, 20 and 40) under both conditions of normal and reverse polarity on peak systolic blood pressure (SBP), heart rate (HR) and ipsilateral splanchnic sympathetic nerve active (sSNA) in Lewis (A) and LPK (B) animals. A biphasic blood pressure response was seen in the LPK, and the two phases are presented separately. Peak effects are shown as absolute change (ΔSBP, ΔHR) or percentage (Δ sSNA) changes from respective baseline value with cathode in both normal and reverse polarities. There was no significant effect of polarity for any of the parameters measured. Values are expressed as mean ± SEM. For Lewis: n = 5 per group for SBP and = 4 per group for sSNA and HR for each polarity. For LPK n = 6 per group.

Supplementary Figure 2: Comparison of polarity (150μA)

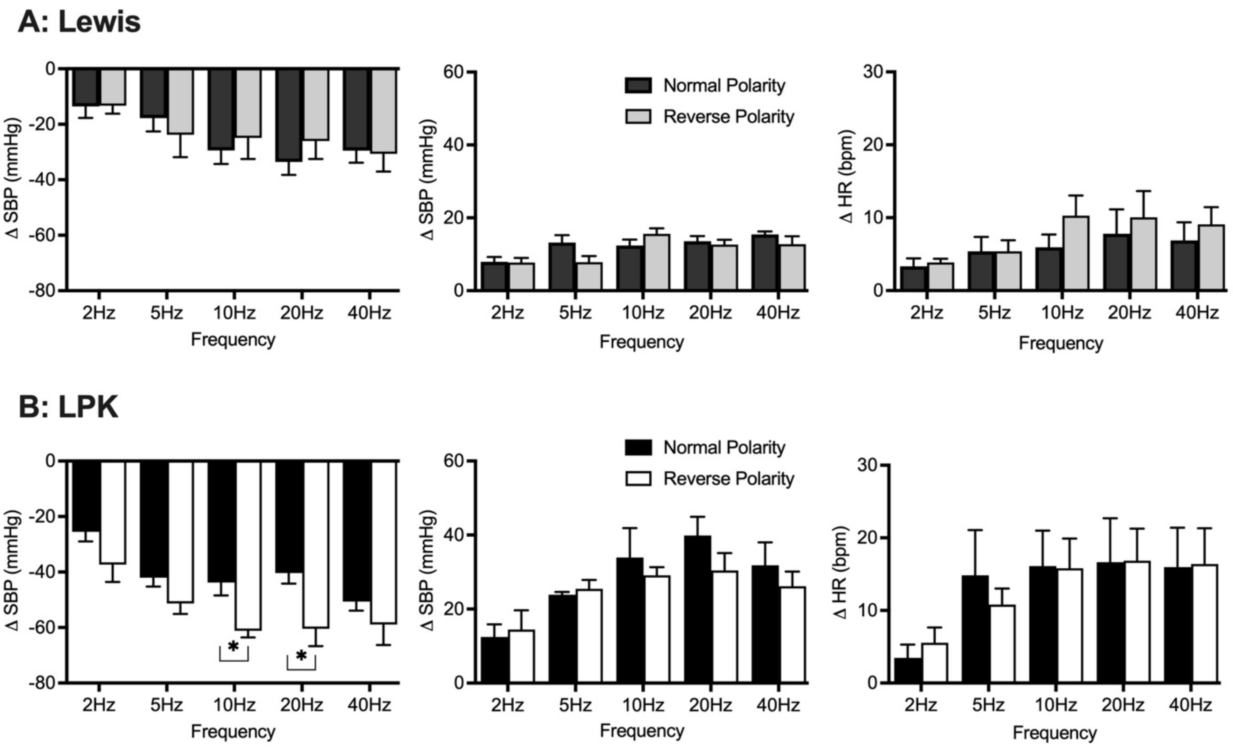

Group data showing comparison of left renal afferent nerve stimulation (150uA) at different Hz (2, 5, 10, 20 and 40) under both conditions of normal and reverse polarity on peak systolic blood pressure (SBP) and heart rate (HR) in Lewis (A) and LPK (B) animals. Peak effects are shown as absolute change (ΔSBP and ΔHR) changes from respective baseline value with cathode in both normal and reverse polarities. A biphasic blood pressure response was seen in response to high-intensity nerve stimulation and the two phases are presented separately. In the Lewis, there was no significant effect of polarity on any parameter. In the LPK there was no significant effect caused by polarity for the pressor or HR parameters however polarity reversal did enhance the depressor responses at 10 and 20 Hz ($P < 0.05$), with an increased magnitude of approximately 10 mmHg compared to normal polarity. Although observed under limited conditions, this finding highlights the potential for refining stimulation parameters in future studies aimed at optimizing neuromodulation strategies for therapeutic applications. Values are expressed as mean ± SEM. (*) = $P < 0.05$. For Lewis n =5 and LPK n = 6 per group.

Supplementary Figure 3: Comparison of recording cuff electrode placement (ipsilateral or contralateral to SNA bipolar recording electrode) on cardiovascular parameters in Lewis animals.

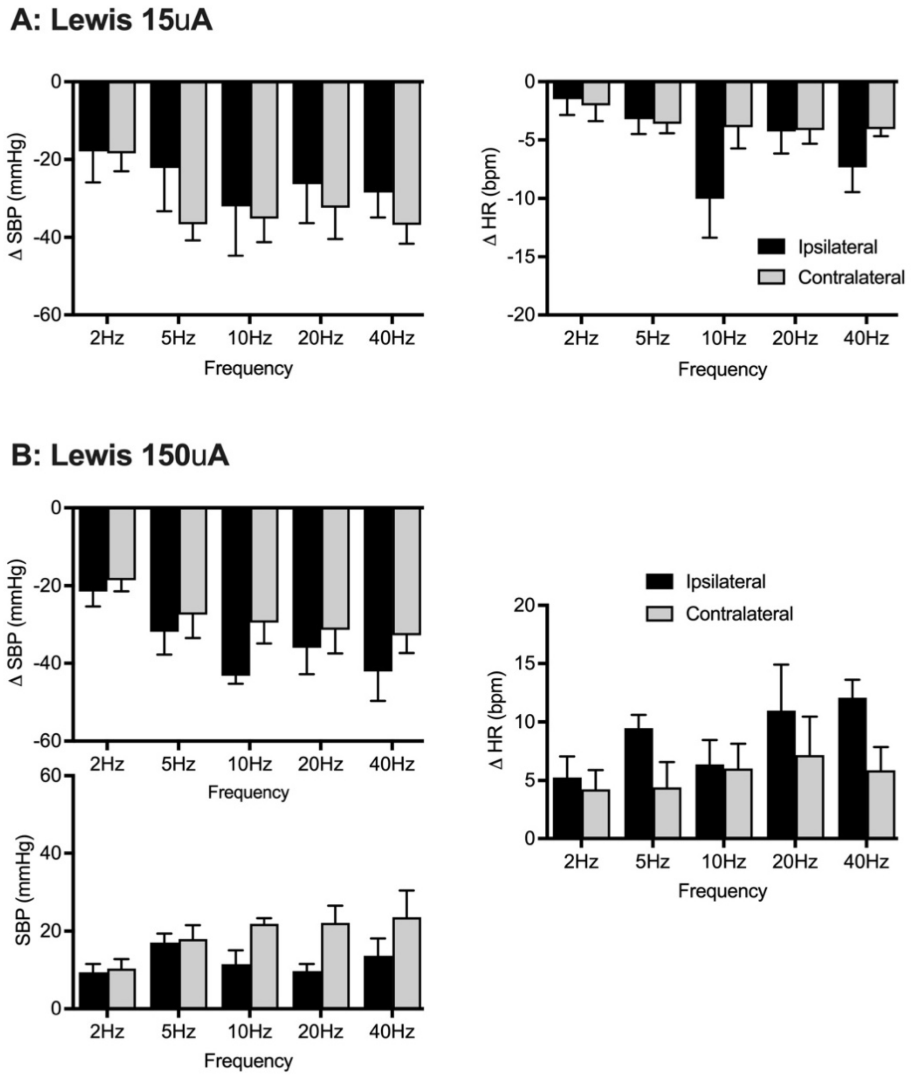

Grouped data from Lewis animals showing effects of renal afferent nerve stimulation (A: low-intensity15 uA and B: high-intensity150uA) at different Hz (2, 5, 10, 20 and 40) comparing response to stimulation using a cuff electrode on the left renal nerve (ipsilateral to recording electrode on left splanchnic nerve) vs. right renal nerve (contralateral to recording electrode on left splanchnic nerve) in Lewis rats on SBP and HR. A biphasic blood pressure response was seen in response to high-intensity nerve stimulation and the two phases are presented separately (B). There was no significant effect of electrode placement on any of the measured parameters. Peak effects are shown as absolute change (ΔSBP, ΔHR) from respective baseline value. Values are expressed as mean ± SEM. n = 3 per group for ipsilateral recordings and 4 per group for contralateral recordings

Supplementary Figure 4: Comparison of recording cuff electrode placement (ipsilateral or contralateral to SNA bipolar recording electrode) on cardiovascular parameters in LPK animals

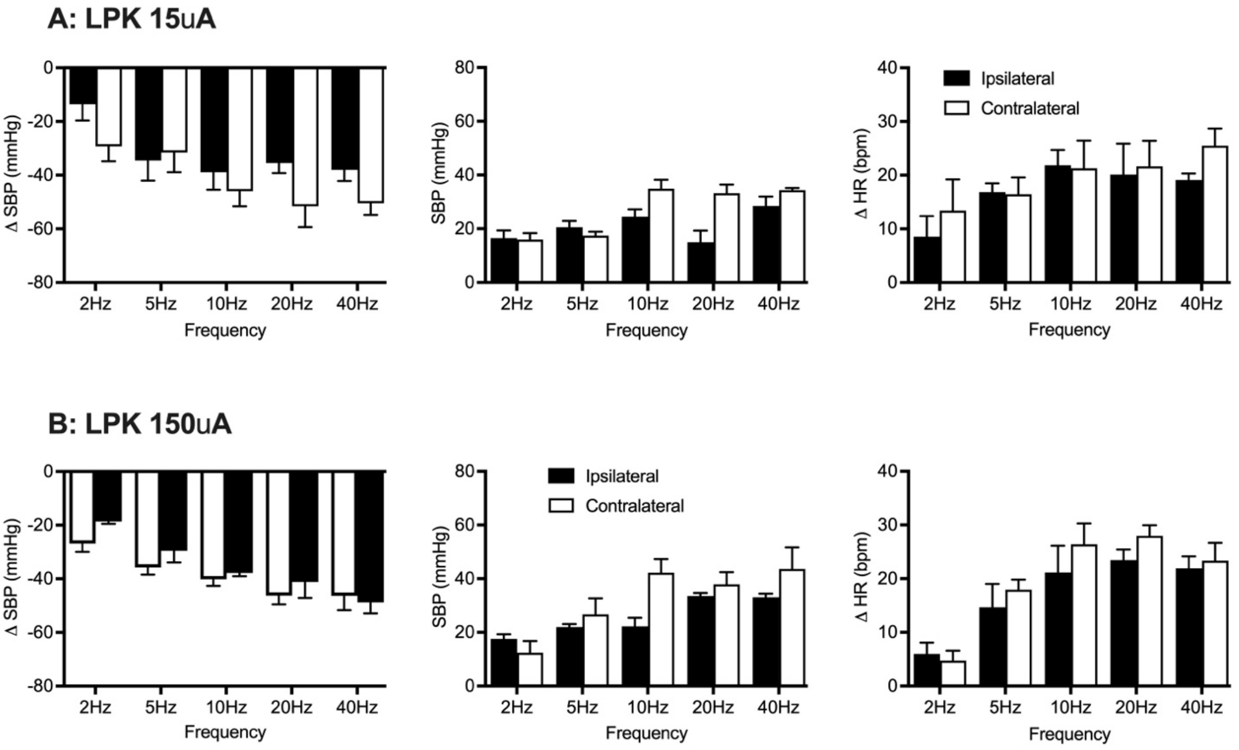

Grouped data from LPK animals showing effects of renal afferent nerve stimulation (A: low-intensity15 uA and B: high-intensity150uA) at different Hz (2, 5, 10, 20 and 40) comparing response to stimulation using a cuff electrode on the left renal nerve (ipsilateral to recording electrode on left splanchnic nerve) vs. right renal nerve (contralateral to recording electrode on left splanchnic nerve) in LPK rats on SBP and HR. A biphasic blood pressure response was seen in response to nerve stimulation and the two phases are presented separately. There was no significant effect of electrode placement on any of the measured parameters. Peak effects are shown as absolute change (ΔSBP, ΔHR) from respective baseline value. Values are expressed as mean ± SEM. n = 4 contralateral, 3 for all other groups.

Supplementary Figure 5: Comparison of recording cuff electrode placement (ipsilateral or contralateral to SNA bipolar recording electrode) on sympathetic response in Lewis and LPK animals

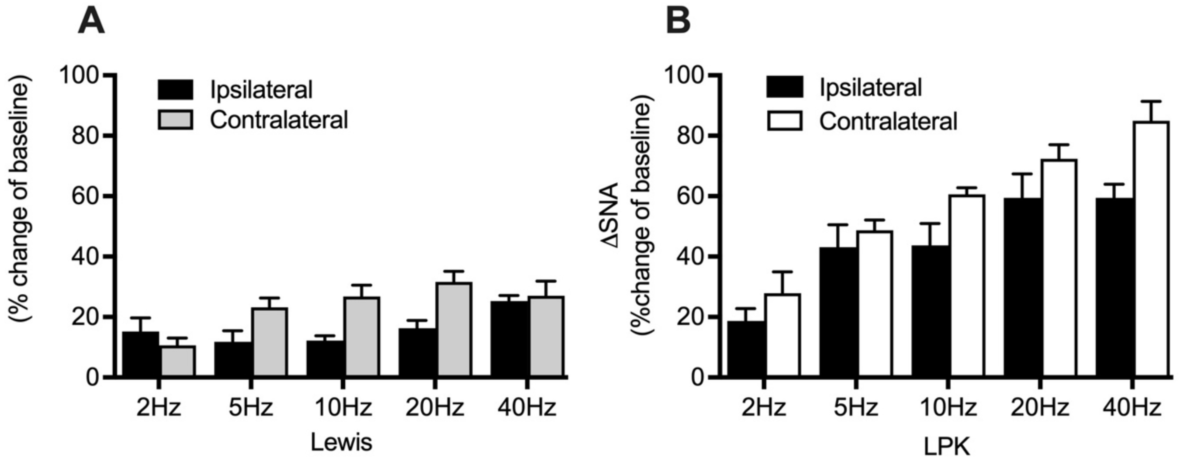

Grouped data showing effects of renal afferent nerve stimulation at low-intensity (15 uA) in Lewis (A) and LPK (B) animals at different Hz (2, 5, 10, 20 and 40), comparing response to stimulation using a cuff electrode on the left renal nerve (ipsilateral to recording electrode on left splanchnic nerve) vs. right renal nerve (contralateral to recording electrode on left splanchnic nerve). There was no significant effect of electrode placement on any of the measured parameters. Peak effects are shown as percentage (Δ sSNA) changes from baseline value. Values are expressed as mean ± SEM. n = 4 contralateral Lewis, 3 for all other groups.

Supplementary Analysis: Comparison of responses using bipolar vs. cuff electrodes

Analysis was undertaken using the data presented in the above figures to compare the responses when using either a bipolar or cuff stimulating electrode. Frequency and electrode type were used as the fixed effects within strain for two-way ANOVA analysis as detailed in the main methods section.

Under low-intensity stimulation parameters (15uA), the splanchnic SNA response in Lewis animals was significantly different (P = 0.02), being greater marginally greater using the bipolar electrode. In the LPK, it was also different (P = 0.006) however the SNA response was greater when using the cuff electrode. The depressor SBP response in both Lewis and LPK was not significantly different with electrode type, nor was the pressor response in the LPK. The tachycardic HR response in the LPK was significantly different (P = 0.0095) but this was only evident as an increased response at 40Hz when using the cuff electrode. In the LPK, the bradycardic response was not different between the electrode types.

When using the high stimulation parameter (150uA), the depressor response in the LPK was different with the different electrodes (P = 0.0273) however post-hoc analysis indicated this was only marginal, being slightly greater at 5Hz only using the bipolar electrode. The depressor SBP response in Lewis was not significantly different with electrode type. The pressor response in the LPK was not significantly different when using the cuff or bipolar electrodes, while in the Lewis, it was different (P = 0.0386) however post-hoc analysis did not show any difference at any specific frequency. Neither the tachycardic HR response in the LPK or the Lewis was significantly different using the different electrode types.

Overall, the responses were in the same direction regardless of electrode type and of very similar magnitude within either strain. The data collected using bipolar electrodes is presented in the main manuscript with the date collected using the cuff electrodes is provided here.

Supplementary Figure 6: Grouped effects of low-intensity stimulation of the renal nerve using a cuff electrode on cardiovascular parameters and sympathetic activity in Lewis and LPK animals

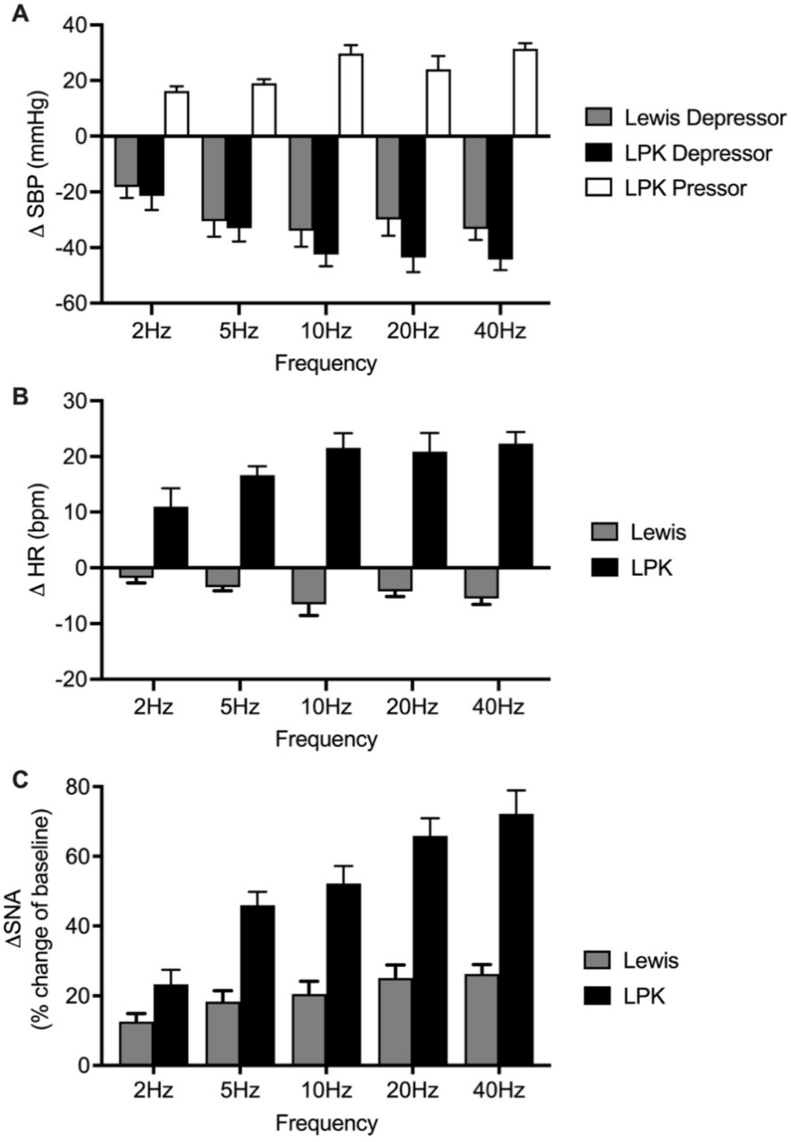

Grouped data showing peak SBP (A; depressor and pressor), HR (B: bradycardia and tachycardia) and sSNA effects produced by left renal afferent nerve stimulation (150uA) at different Hz (2, 5, 10, 20 and 40) in Lewis and LPK rats using the cuff electrode. Peak effects are shown as absolute change (A: ΔSBP, B: ΔHR) or percentage (C: ΔsSNA) changes from respective baseline value. sSNA data includes combined data sets after recording both contralateral and ipsilateral to the stimulating cuff electrode.

In the Lewis rat, low-intensity stimulation elicited a significant depressor response, bradycardia, and sympathoexcitation. There was a frequency effect on the depressor SBP response ($P < 0.001$), with the peak observed at 5 Hz, followed by a subsequent plateau (A). There was no frequency effect on HR ($P = 0.0840$; B). The was a frequency effect on sympathoexcitation ($P < 0.001$) with the maximal sSNA response occuring at 5 Hz followed by a plateau (C).

In the LPK rat, low-intensity renal afferent nerve stimulation triggered a biphasic BP response (depressor followed by pressor), tachycardia, and sympathoexcitation. Both phases of the BP response were frequency-dependent ($P < 0.05$) with a peak response at 5 Hz then reaching a plateau. The tachycardic response was frequency dependent ($P = 0.0043$) noting the only post-hoc difference was between 2 and 10 Hz. Sympathoexcitation was frequency dependent ($P < 0.0001$; C) peaking at 20 Hz. Values are expressed as mean ± SEM (n = 7 Lewis, 6 LPK). Statistical results for post-hoc frequency analysis are provided in Supplementary Data 1.

When comparing responses using cuff electrodes in the Lewis and LPK groups using low intensity stimulation, the latter demonstrated a significantly greater sympathoexcitatory activity ($P < 0.0001$; C). Cardiovascular hemodynamics following low frequency stimulation were not compared between strains given that they elicited divergent responses.

Supplementary Figure 7: Grouped effects of high-intensity stimulation of the renal nerve using a cuff electrode on cardiovascular parameters in Lewis and LPK animals

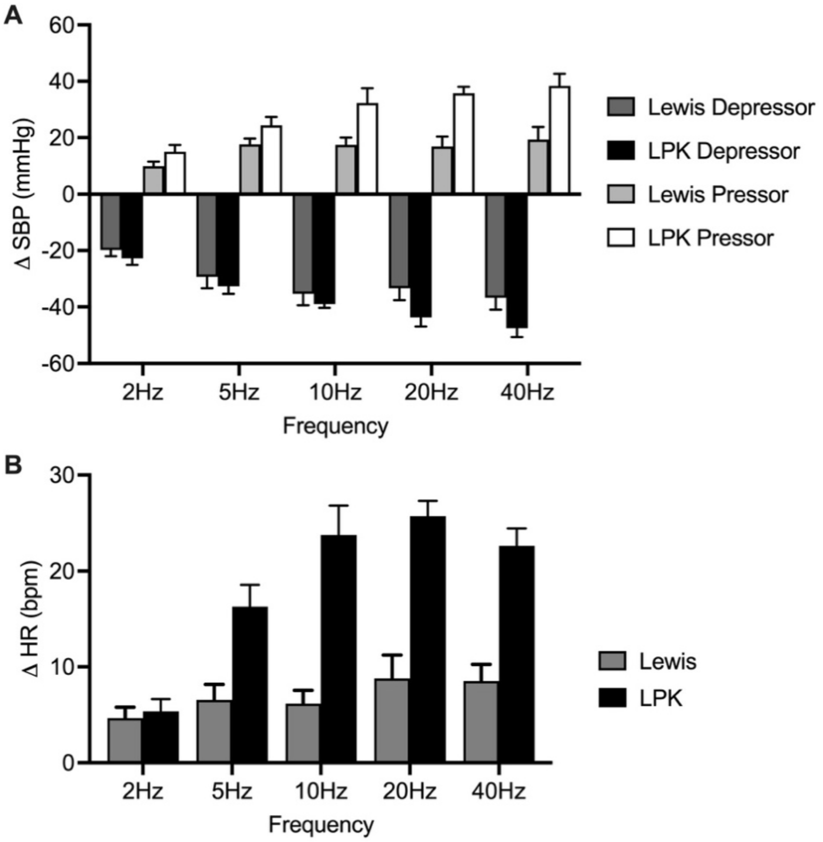

Grouped data showing peak SBP (depressor and pressor) and HR (bradycardia and tachycardia) effects produced by left renal afferent nerve stimulation (150uA) using a cuff electrode at different Hz (2, 5, 10, 20 and 40) in Lewis and LPK rats. Peak effects are shown as absolute change (A: ΔSBP, B: ΔHR) from respective baseline value.

High-intensity stimulation induced a biphasic BP response and tachycardia in the Lewis rat. The depressor response was significantly impacted by stimulation frequency ($P < 0.0001$; A), reaching maximal effect at 5 Hz then plateauing. The pressor and HR responses were not influenced by frequency (A, B).

At high-intensity stimulation in the LPK rat, a biphasic BP response was again appreciated (A). Both phases were frequency-dependent ($P < 0.0001$), with maximal effect at 5- 10 Hz. HR was also modulated by frequency under these conditions (B; $P < 0.0001$), peaking at 10 Hz.

When comparing responses using cuff electrodes in the Lewis and LPK groups using high intensity stimulation, both groups demonstrated comparable changes in the depressor phase of the BP response (A; $P = 0.1136$), but there was a heightened pressor response in the LPK group (A; $P = 0.0053$). LPK animals also demonstrated more marked tachycardia in response to high-intensity stimulation (B; $P = 0.0001$). Statistical results for post-hoc frequency analysis are provided in Supplementary Data 1.

Values are expressed as mean ± SEM and (n = 7 Lewis, 6 LPK).

Supplementary Figure 8: Baseline afferent renal nerve activity

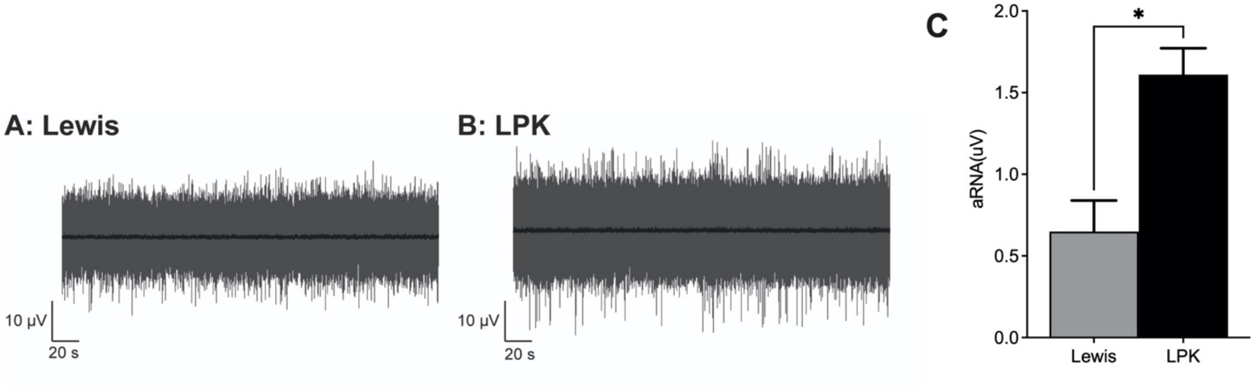

Panels A and B provide representative traces of raw data of the renal nerve demonstrating baseline afferent renal nerve (aRNA µV) discharge recorded using a bipolar electrode from Lewis (A) and LPK (B) animals. Panel C provides the group data after rectification, smoothing and subtraction of background (mean ± SEM).

Supplementary Figure 9: Response of splanchnic sympathetic nerve to renal nerve stimulation

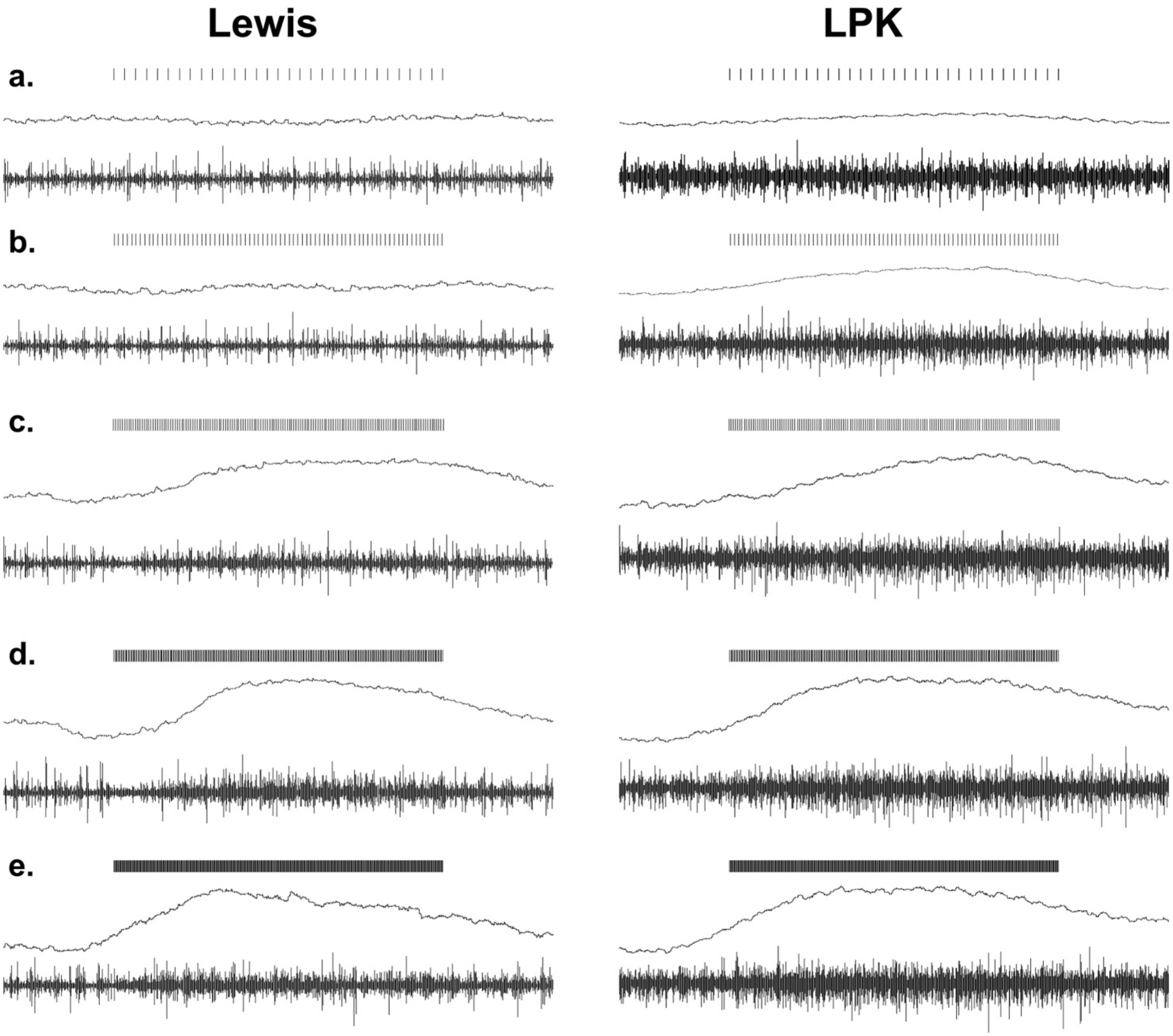

Representative expanded traces of processed (rectified and integrated) and raw traces of ipsilateral splanchnic sympathetic nerve activity (sSNA) responses during left renal nerve stimulation (15 µA) using a bipolar electrode at different stimulation frequencies (2, 5, 10, 20, and 40 Hz) in Lewis (left panel) and LPK (right panel) rats. Each trace includes 5 seconds of baseline activity, 15 seconds of stimulation as indicated by hatched bars, and 5 seconds of post-stimulation recording. Panels (a–e) correspond to the different stimulation frequencies: (a) 2 Hz, (b) 5 Hz, (c) 10 Hz, (d) 20 Hz, and (e) 40 Hz. These expanded traces provide a clearer visualization of sSNA burst activity, as well as its modulation by renal nerve stimulation.
